# Conformational Dynamics of Dry Lamellar Crystals of Sugar Based Lipids: An Atomistic Simulation Study

**DOI:** 10.1371/journal.pone.0101110

**Published:** 2014-06-30

**Authors:** Vijayan ManickamAchari, Richard A. Bryce, Rauzah Hashim

**Affiliations:** 1 Department of Chemistry, University of Malaya, Kuala Lumpur, Malaysia; 2 Manchester Pharmacy School, University of Manchester, Manchester, United Kingdom; 3 Kavli Institute of Theoretical Physics China, Chinese Academy of Sciences, Beijing, China; Jacobs University Bremen, Germany

## Abstract

The rational design of a glycolipid application (e.g. drug delivery) with a tailored property depends on the detailed understanding of its structure and dynamics. Because of the complexity of sugar stereochemistry, we have undertaken a simulation study on the conformational dynamics of a set of synthetic glycosides with different sugar groups and chain design, namely dodecyl *β*-maltoside, dodecyl *β*-cellobioside, dodecyl *β*-isomaltoside and a C_12_C_10_ branched *β*-maltoside under anhydrous conditions. We examined the chain structure in detail, including the chain packing, *gauche*/*trans* conformations and chain tilting. In addition, we also investigated the rotational dynamics of the headgroup and alkyl chains. Monoalkylated glycosides possess a small amount of *gauche* conformers (∼20%) in the hydrophobic region of the lamellar crystal (L_C_) phase. In contrast, the branched chain glycolipid in the fluid L_α_ phase has a high *gauche* population of up to ∼40%. Rotational diffusion analysis reveals that the carbons closest to the headgroup have the highest correlation times. Furthermore, its value depends on sugar type, where the rotational dynamics of an isomaltose was found to be 11–15% and more restrained near the sugar, possibly due to the chain disorder and partial inter-digitation compared to the other monoalkylated lipids. Intriguingly, the present simulation demonstrates the chain from the branched glycolipid bilayer has the ability to enter into the hydrophilic region. This interesting feature of the anhydrous glycolipid bilayer simulation appears to arise from a combination of lipid crowding and the amphoteric nature of the sugar headgroups.

## Introduction

Glycolipid surfactant is cheap, non-ionic, and biodegradable [1] with a wide range of applications from emulsifiers, delivery systems to solubilization and extraction of membrane proteins [2]. Other features like low toxicity, low immunogenicity and specific sugar-cell recognition have broadened further the scope of interest in this material, including many fundamental studies. Glycolipids also exist naturally as a minor component in cell membranes, such as those found in Gram positive bacteria [3] and in the photosynthetic membranes of green plants [4]. But extracting them from natural products is challenging, and not cost effective. Therefore, highly pure synthetic glycolipids are usually used for applications and research studies. The amphiphilic nature of these lipids, both hydrophilic and hydrophobic, enable them to self-assemble into micelles, hexagonal phases, bilayers and other complex three dimensional cubic structures, generally in polar solvent, mainly water or even in dry conditions, as described recently [5]–[10]. Amongst these, the two-dimensional lipid bilayer is more important for its close resemblance to that of a phospholipid which is usually used to model the plasma- membrane [11]. Within a simple lipid bilayer structure, there exists detailed variation leading to a variety of mesophases, due to effects such as solvent type, concentration and temperature. For example, a hydrated bilayer is classified as a liquid crystalline (L_α_) phase, in which the alkyl chains are disordered above the main transition temperature, T_C_, while below that, the system assumes a gel phase (L_β_), where the extended lipid chains tilt to the normal of the bilayer. At a much lower temperature, the bilayer forms a lamellar crystalline phase (L_C_) where the extended lipid chains now have much lower tilting angles relative to those in the gel phase [12]. At zero or at a very low hydration, most of the lipids form a lamellar crystalline (L_C_) phase [6], [13], [14]. Usually these lamellar phases show both long and short range order similar to a true crystal [14]. Meanwhile, other factors such as the molecular geometry, chain design and headgroup size influence the occurrence of these phases [12]. For example, monoalkylated glycolipids, at room temperature and under dry conditions, usually exist in the lamellar crystal phase (L_C_) [15].

Bilayers of phospholipids have been studied extensively [16], [17] compared to those of glycolipids, primarily due to the challenging task of dealing with the sugar's stereochemistry [5]. Thus, to unravel the glycolipid's complexity, a systematic structure property relationship study is useful. Investigation of these systems in the anhydrous state is also necessary to understand the detailed behavior of individual lipids uncomplicated by the presence of solvent. Recently some synthetic glycolipids bilayers have been studied in dry as well as hydrated forms [18], [19], including dodecyl *β*-maltoside (*β*Mal-C_12_) which exists in an L_C_ phase over a temperature range of 20–80°C [19]. Within this range, this phase has low thermal fluctuation and may give interesting properties different from phospholipids under anhydrous conditions [20]. However, glycolipids are highly hygroscopic; hence, it is difficult to have a completely pure and dry substance under normal experimental conditions.

**Figure 1:**
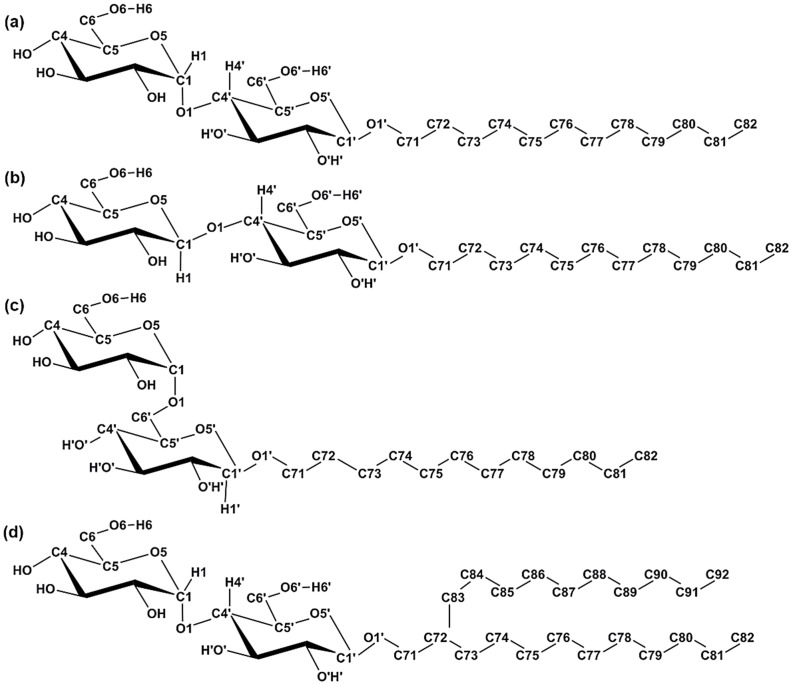
Glycosides used in the simulation: (a) *β*Mal-C_12_ (b) *β*Cel-C_12_ and (c) *β*IsoMal-C_12_ (d) *β*BCMal-C_12_C_10_. Starting from glycosidic oxygen, each glycolipid's main alkyl chain (*sn-1*) is labeled from C71 to C82 and the branched chain (*sn-2*) from C83 to C92. Here, for clarity we have reduced the atoms' labels on the sugar, a slight modification from the standard nomenclature according to IUPAC given in Figure 1 of [22].

Considering these points, we have undertaken a molecular dynamics simulation study using full atomistic resolution on four synthetic glycolipids namely, dodecyl *β*-maltoside (*β*Mal-C_12_, Figure 1a), dodecyl *β*-cellobioside (*β*Cel-C_12_, Figure 1b), dodecyl *β*-isomaltoside (*β*IsoMal-C_12_, Figure 1c) in the L_C_ phase and a branched chain maltoside (*β*BCMal-C_12_C_10_, Figure 1d) in the L_α_ phase. The liquid crystalline fluid phase (L_α_) is known to possess complex motions covering a wide range of time scales. Motions in the picosecond range include chemical bond vibration and torsional oscillation, while rotations, *gauche*/*trans* isomerization, and lipid wobbling occur over nanoseconds. Slow dynamics from milliseconds to hours are usually associated with the in-plane lateral diffusion, undulation and flip-flop of lipids [21]. In addition the amphoteric nature of sugar makes the dynamics of these carbohydrate liquid crystal phases even more intriguing and challenging. Therefore, we have extended our investigation into the conformational dynamical behavior of glycolipids in an anhydrous form to understand how these properties relate to the sugar stereochemistry and the lipid chain architecture.

## Materials and Methods

### Lipid Model Construction

Using IUPAC nomenclature, [23], [24] the relative orientation of a reducing and a non-reducing sugar in disaccharides of *β*Mal-C_12_ and Cel-C_12_ are represented by the dihedral angles (Φ and Ψ) of H1-C1-O1-C4' and C1-O1- C4'-H4' respectively. For an isomaltoside (*β*IsoMal-C_12_), these are Φ (O5-C1-O1-C6'), Ψ (C1-O1-C6'-C5') and ω (O1-C6'-C5'-C4'). Initial values of Φ, Ψ and ω for these glycosides were obtained from the literature [23], [25], [26] (for details see Table S1). Using this information, the starting monomeric structures of these glycolipids were modeled and geometrically optimized using the HyperChem package [27]. A single monolayer with an 8×8 lipids was built and was geometry optimized. This monolayer was used to form a bilayer with the tail group of the lipids pointing towards each other and headgroups facing the opposite directions. Since there is no single crystal structure information available for *β*Mal-C_12_ [19], we have used crystal builder option in HyperChem to build the single bilayer. Then, the bilayer was replicated to form a second bilayer to give a simulation cell with a total of 256 lipids. The double bilayer system was used to resemble closely the experimental (lamellar) conditions [28]. A recent study by Abe *et al*. supports the assertion that the alkyl chains of glycolipid in L_C_ phase are not perpendicular to the bilayer normal but tilted slightly [29]. Additionally, Abeygunaratne *et al*. also provide information on the tilt glycolipid structure in the smectic C liquid crystals by using optical microscopic and the electric polarization experimental methods [30]. Taking these observations into consideration, in our model, we have pre-tilted the lipid chains at about 15° to the bilayer normal. At the same temperature, the branched chain glycolipids give an anhydrous fluid L_α_ phase [31].

### Simulation Details

MD simulations were performed using the AMBER molecular dynamics package [32] with force field parameter sets GLYCAM_06d [33] and *ff99* [32] for the lipid head and tail respectively. An equilibration procedure was applied, involving restrained energy minimizations on tail and headgroup moieties separately. The system was then heated gradually over 2 ns from 0 to 27°C in the *NVT* ensemble using the Andersen thermostat (τ_TP_ = 0.5 ps) [34]. Upon reaching 27°C, the restraints were reduced on the lipids stepwise in the *NpT* ensemble using weak anisotropic pressure coupling, τ_P_ = 2 ps. A time step of 1 fs was used with the SHAKE algorithm [35], [36] to constrain covalent bonds involving hydrogen. Periodic boundary conditions were applied to the simulation box and long-range electrostatics for non-bonded interactions were treated using the particle mesh Ewald method [37], [38]. We reprise our earlier 150–180 ns simulations of these systems [22], performing replicate simulations of 200 ns within the *NpT* ensemble at 27°C, but permitting fully anisotropic pressure scaling, as opposed to the isotropic conditions used previously. The trajectory coordinates were archived every 5 ps. As bilayers need at least 20 ns run to equilibrate [39], the last 160 ns of each 200 ns simulation was used for analysis. These simulations were performed with the GPU-accelerated version of the *pmemd* module.

### Analysis

#### Area per head group and local density profile

Conventionally, the stability of the four bilayer assemblies, *β*Mal-C_12_, *β*Cel-C_12_, *β*IsoMal-C_12_, and *β*BCMal-C_12_C_10_ were monitored over the entire simulation by observing properties such as the surface area per lipid at the interface (*A*) and the local density profile (LDP) [39], [40]. The former (*A*) was estimated by applying a standard procedure, dividing the area of *x*–*y* plane by the number of lipids present in a leaflet (Figure S2) [41], while, the latter was obtained from the average of five 40 ns block averages of the 200 ns simulation (Figure S3). This block-averaged LDP profile is useful in evaluation of the convergence of the simulation with respect to the distribution of sugar and chain residues within the simulated system [39].

### Radial distribution function

The packing of alkyl chains in the hydrophobic region was determined by calculating the radial distribution function (RDF) for selected carbon atoms on a lipid to the neighbouring lipids [42]. The RDF function 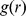 is obtained from
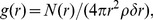
(1)where, 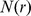 is the number of selected atoms between distance 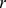 and 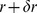 from the reference atom. 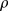 is the number density [43]. We choose three carbon atoms, namely C72, C76 and C81, along a lipid chain from each monoalkylated glycosides corresponding to: near sugar moiety, middle and near to methyl group. The related RDFs were calculated using the *ptraj* module in AMBER.

#### Gauche and trans population distribution

Alkyl chains may contain several methylene groups, whose conformations [44] determine the detailed phase behavior [45]. These conformations were analysed by calculating the probability, 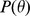 of the *gauche*/*trans* conformers, defined as, 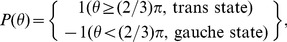
(2)where *θ* is the dihedral angle formed by four consecutive carbons along the alkyl chain [17]. The *ptraj* module in AMBER was used to evaluate this probability function.

#### Autocorrelation function

To understand the dynamics of glycolipids in the self-assembled system, rotational motions of the alkyl chain and sugar headgroup have been examined separately. For the headgroup, we selected three rotational systems namely, the reducing sugar (*ring2*), non-reducing sugar (*ring1*) and the combined sugar rings (*ring12*), represented by the appropriate unit vectors, 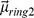 from C1 to C4, 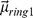 from C1' to C4', and 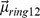 from C1' to C4 respectively (see Figure S1(a)). For the rotational diffusion of the chain region, the C–H vectors along the alkyl chain were chosen. Using these vectors and *ptraj* module from AMBER the correlation times for the various motions were estimated using the second rank reorientational autocorrelation functions, 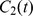 defined by [46]:
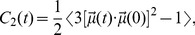
(3)where 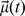 is a unit vector of the chosen rotational mode. 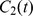 was evaluated for each lipid over the production trajectory and averaged over the number of lipids within the system. The average 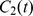 was fitted to a single-exponential function; from which the correlation time was obtained by integration using the trapezoidal rule. The standard deviation of 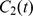 was also determined to within the range of ±0.5 ns.

## Results

### Stability and structural properties of bilayers

In order to evaluate the equilibration of the four glycolipid bilayer systems, the time evolution of the surface area per lipid at the interface (*A*) and the local density profiles (LDP) were assessed over the 200 ns simulation (Figures S2 and S3 respectively). The four bilayer assemblies, *β*Mal-C_12_, *β*Cel-C_12_, *β*IsoMal-C_12_, and *β*BCMal-C_12_C_10_, remained intact over the simulations and achieved equilibrium with respect to *A* and LDP by 40 ns. In the subsequent analyses, we therefore take the last 160 ns of each trajectory. The average area per lipid (*A*) and the *d*-spacing from the LDPs during the production stage are given in Table 1. Compared to the reported values in [22], in our replicate simulations here, *A* for the four glycolipid systems are similar to within the error; the *d*-spacing differ by only about 3–16%. These variations could be attributed in part to the slight difference in simulation methodology: as opposed to the isotropic pressure scaling used in our previous study, here we apply fully anisotropic pressure scaling throughout the simulation, following the work of Doxastakis *et al*. [20] on simulating the melting of phospholipid membranes under anhydrous conditions.

**Table 1:** Average values at 27°C of the simulated area per lipid, *A* and the bilayer spacing, *d*.

Lipids	Area per lipid, *A*/Å^2^	Bilayer spacing, *d*/Å	Peak value of Chain tilt angle, θ°
*β*Mal-C_12_	39.4±0.2 (38.8±0.2)	32.2±0.1 (32.9±0.5)	18±1 (18)
*Experiment* [19]	43 (20°C)	33.5 (20°C)	
		41.5 Å (at 80°C)	
*β*Cel-C_12_	39.2±0.2 (38.7±0.1)	34.1±0.1 (32.3±0.2)	15±1 (13)
*β*IsoMal-C_12_	49.1±0.3 (48.6±0.2)	22.9±0.2 (26.6±0.6)	42, 59±3 (26)
*β*BCMal-C_12_C_10_	50.1±0.3 (51.9±0.2)	41.9±0.2 (36.1±0.2)	24, 156±3 (s*n-1*) (27)
*β*BCMal-C_12_C_10_			24, 156±3 (s*n-2*) (30)
*Experiment* [47]			
*β*Mal-C_12_C_8_	53 (25°C)	36.2 (25°C)	
*β*Mal-C_14_C_10_	58 (25°C)	36.9 (25°C)	

(Values in parenthesis are from [22]).

### Alkyl chain packing

**Figure 2:**
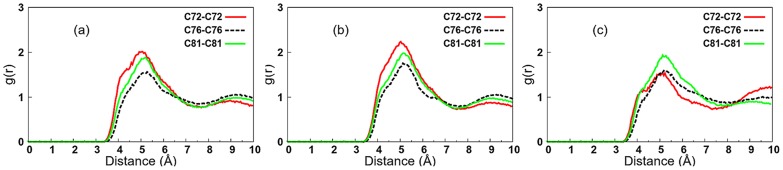
Radial distribution function for three carbon atoms along the alkyl chain for monoalkylated lipids: (a) βMal-C_12_ (b) βCel-C_12_ and (c) βIsoMal-C_12_.

**Figure 3:**
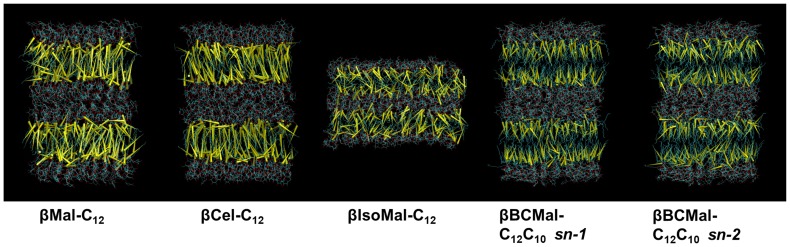
Snapshot of chain vectors between midpoints of C71–C72 and C80–C81 for monoalkylated lipids and chain *sn-1*. For chain *sn-2*, the vector is defined between midpoints of C71–C72 and C90–C91.

The packing within the chain region for the *β*Mal-C_12_, *β*Cel-C_12_ and *β*IsoMal-C_12_ was assessed by computing the radial distribution function, giving the particle density variation as a function of distance from a reference particle, selected to be the carbon atoms C72, C76, and C81 along the hydrophobic chain. For all the three monoalkylated systems, the principal peaks in the RDF are around 5.1, with the errors for *β*Mal-C_12_ and *β*Cel-C_12_ within ±0.2 Å while for *β*IsoMal-C_12_ ±0.5 Å, (Figure 2). The errors were estimated using 20 ns block averages. The RDF of *β*IsoMal-C_12_ shows a broader first maximum peak for all the three carbons along the alkyl chain (Figure 2c) compared to *β*Mal-C_12_ and *β*Cel-C_12_ (Figure 2a,b). While the peak locations are nearly the same for all systems, the broadening around the peak in the isomaltoside RDF is considerably larger than for the others, suggesting that the *β*IsoMal-C_12_ chains are less ordered compared to the other two monoalkylated lipids, despite being in the same phase. Furthermore, for isomaltoside, the second last carbon, C81, is more structured, while in the maltoside and cellobioside, it is the carbon closest to the headgroup, C72, which is more structured. To examine this observation visually, we have superimposed a vector (in yellow) for every chain in a given configuration snapshot (Figure 3). This figure points to a tighter packing in *β*Cel-C_12_ compared to *β*Mal-C_12_, while for *β*IsoMal-C_12_ the chain vectors are more randomly orientated, due to an increase in lateral area related to the α(1–6) glycosidic linkage. It also appears that some of the *sn-2* chain vectors of the branched chain maltoside appear to protrude into the sugar region (Figure 3), while such protrusions are less obvious for its *sn-1* vectors. The protrusion of the branched chain maltoside will be discussed in detailed later.

### *Gauche*-*trans* population for alkyl chain

**Figure 4:**
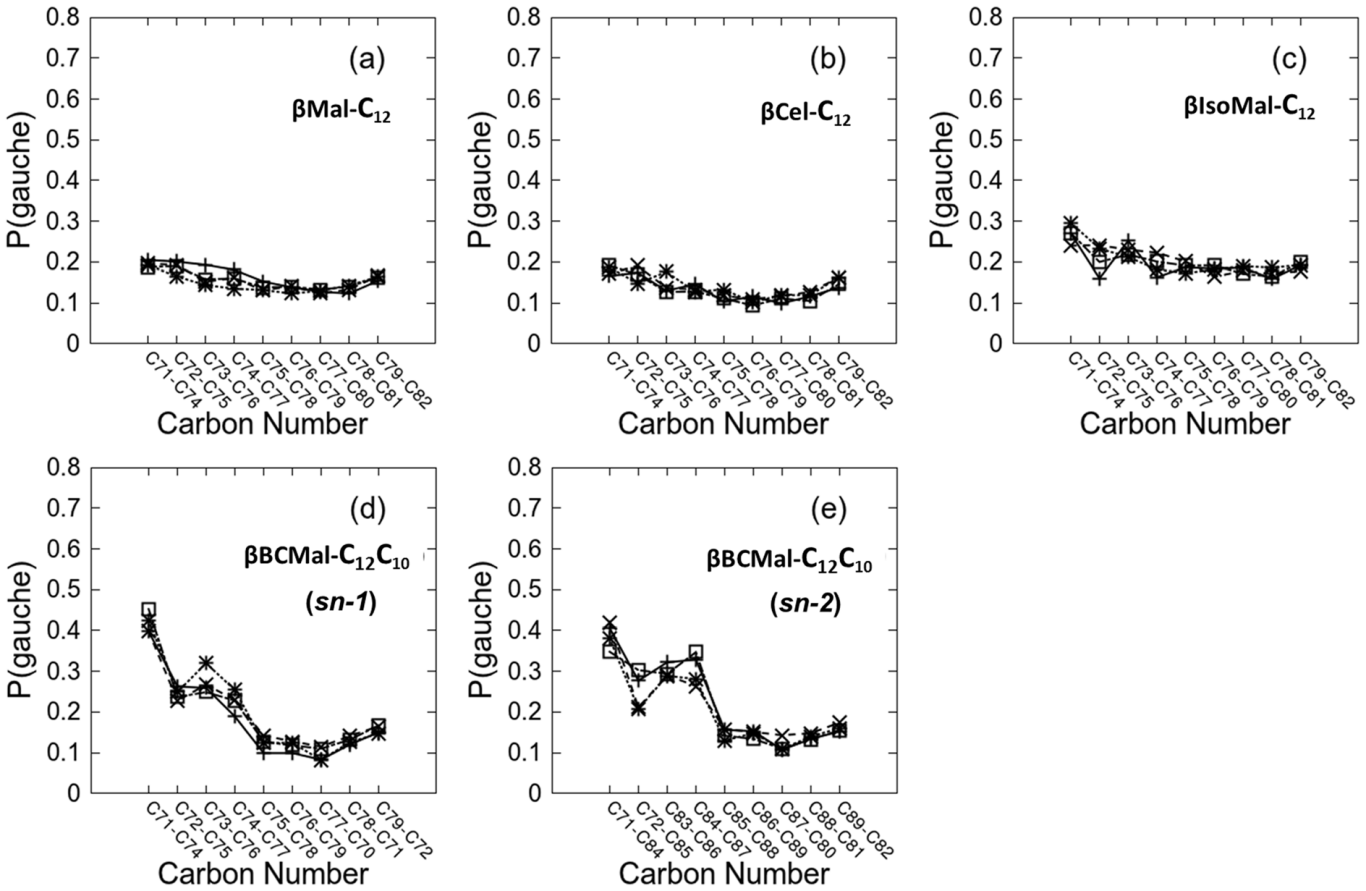
Fractional *gauche* population in, *P*(*gauche*), for dihedral angles between carbons in the alkyl chains. The dihedral angle label, for example C71–C74 represent C71–C72–C73–C4 following alkyl chain numbering in Figure 1. (a) *β*Mal-C_12_, (b) *β*Cel-C_12_, (c) *β*IsoMal-C_12_, (d) *β*BCMal-C_12_C_10_(*sn-1*), and (e) *β*BCMal-C_12_C_10_(*sn-2*). First lipid layer (□), second layer (×), third layer (+), and fourth layer (*).

The *gauche* populations were computed for the lipid chains for *β*Mal-C_12_, *β*Cel-C_12_ and *β*IsoMal-C_12_ in their L_C_ phase (Figure 4a-c) and for *β*BCMal-C_12_C_10_ in the L_α_ phase (Figure 4d-e). We find the monoalkylated glycolipids possess a low proportion of *gauche* conformations relative to *trans* conformations; for example, the average *gauche* populations for *β*Cel-C_12_ and for *β*Mal-C_12_ are 14±3% and 16±3%, respectively (Figure 4a,b). To within error, these results show that both *β*Cel-C_12_ and *β*Mal-C_12_ have similar *gauche* populations despite the fact that the former has two glucose units equatorially connected at the C1–O1 bond, while in the latter, the two units are axially connected (see Figure 1). The closely packed headgroups possibly induce the chains to align closer to each other, restricting isomeric rotation, and leading to fewer kinks or bending along the chain. *β*IsoMal-C_12_ has a higher predicted *gauche* population (20±4%); its larger volume and area per lipid [22], correlates with more freely rotating and flexible chains. For the branched glycolipid, *β*BCMal-C_12_C_10_ (in the L_α_ phase), although it has the same headgroup as *β*Mal-C_12_ (in the L_C_ phase), the branching at C72 (Figure 1d) gives an overall increase in volume and headgroup area *A*. This corresponds to a significant increase in *gauche* population in the branched chain *sn-1* (Figure 4d). Additionally, the *sn-2* chain possesses a *gauche* population ranging from 40% (close to the sugar head) to 10% (at the tail end). The *gauche* profiles for *sn-1* and *sn-2* (Figure 1d and e) look similar in general. However, upon closer examination within each leaflet, close to the headgroup, the first four *gauche* fractions differ slightly with no definite pattern. This slight differentiation may cause unequal flexibility or mean curvature between leaflets leading to bilayer asymmetry [48], [49].

### Rotational motion

Dynamics in the L_C_ phase is understandably limited compared to the L_α_ phase. Within this more restricted dynamical landscape, we seek to examine if there are dynamical features that differ as a function of stereochemical changes of glycolipid in the self-assembled system. To this end, we examine the rotational modes of the alkyl chain and sugar headgroups.

### Alkyl chain

**Figure 5:**
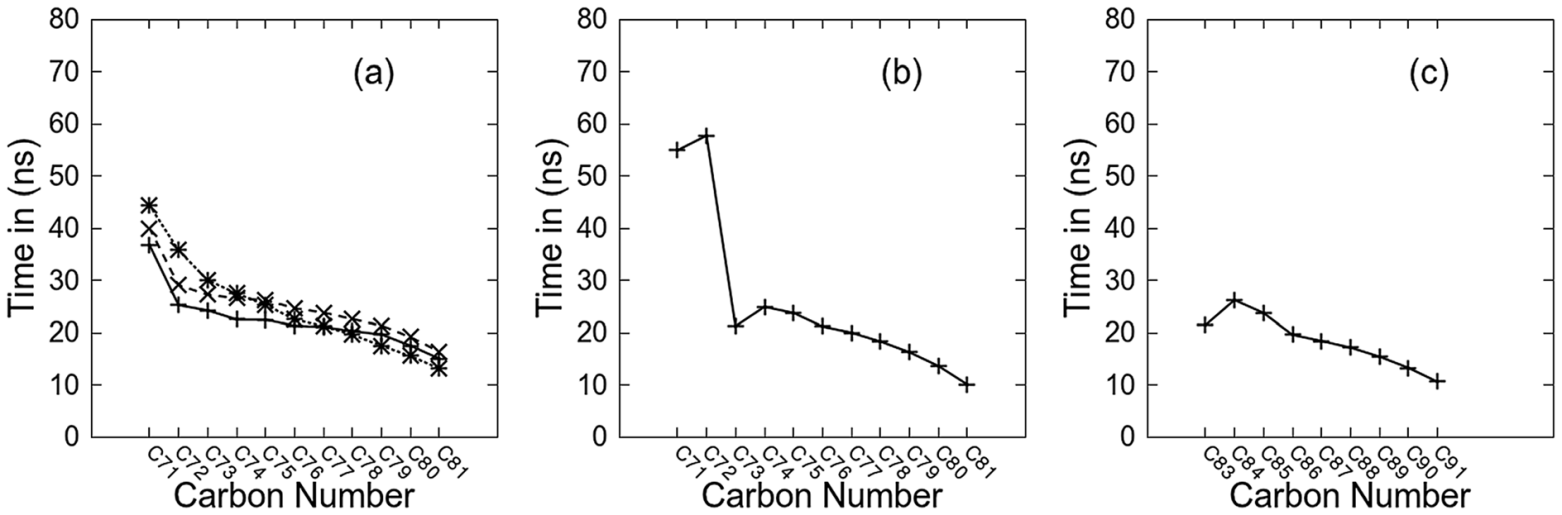
Correlation times as a function of each C–H vector along lipid alkyl chains are shown. The labeling of carbon atoms follows the naming convention as in Figure 1. (a) shows the correlation times for monoalkylated glycolipids, *β*Mal-C_12_ (**+**), *β*Cel-C_12_ (**×**), and *β*IsoMal-C_12_ (*). (b) and (c) show correlation times for chains *sn-1* and *sn-2* respectively for *β*BCMal-C_12_C_10_.

Generally, rotational diffusion in the anhydrous glycolipid systems is slow (on the nanosecond timescale) for the alkyl chain C–H vectors in the chain region, unlike those reported for hydrated phospholipid bilayers in an L_α_ phase [46], [50]. The general appearance of the rotational autocorrelation functions 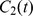 is similar for all the systems (Figure S5). However, upon closer scrutiny (Figure 5), the correlation times of the C–H vectors next to the sugar group are higher than those vectors further away from the sugar group. This observation agrees with a recent study on an anhydrous DPPC (dipalmitoylphosphatidylcholine) bilayer [20] suggesting the tail region closer to the headgroup is mainly experiencing vibrations, with only a small number of conformation transitions.

When compared to the chains of *β*Cel-C_12_ and *β*Mal-C_12_, the alkyl chain of *β*IsoMal-C_12_ shows slightly higher 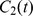 values for carbons close to the headgroup (Figure 5). For example, C71 has a correlation time about 45 ns compared to 40 and 38 ns for *β*Cel-C_12_ and *β*Mal-C_12_ respectively (Figure 5). However, towards the end of the tail, the alkyl chain of *β*IsoMal-C_12_ has slightly lower correlation times compared to *β*Cel-C_12_ and *β*Mal-C_12_.Thus, the alkyl chain carbons of *β*IsoMal-C_12_ appear to be more restricted in rotational motion involving carbons near the headgroup compared to the tail; the latter effect correlates with the chain disorder and partial interdigitation of *β*IsoMal-C_12_ compared to the other monoalkylated lipids (Figure 3). In the *β*BCMal-C_12_C_10_ system, the first two carbons in *sn-1* chains show higher correlation time compared to those in the monoalkylated systems. On the other hand the rest of the carbons behave similarly, as those in the single chain lipids, including *sn-2*.

### Sugar head

**Figure 6:**
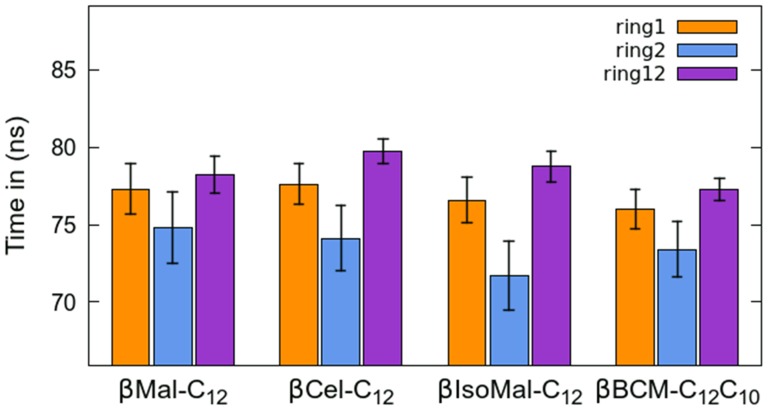
Average correlation time of sugar headgroup of lipids: reducing sugar (*ring1*); non-reducing sugar *(ring2)*; and combination of *ring1* and *ring2* (*ring12*).

A disaccharide unit contains two simple sugars connected via a flexible glycosidic bond, which allows each sugar moiety to rotate within its vicinity. We have determined the rotational diffusion of the sugars at the headgroup region using equation 3. Figure S6 shows the autocorrelation profiles for the non-reducing sugar (*ring1*), reducing sugar (*ring2*) and both sugars together (*ring12*), while Figure 6 gives the corresponding correlation times.

**Figure 7:**
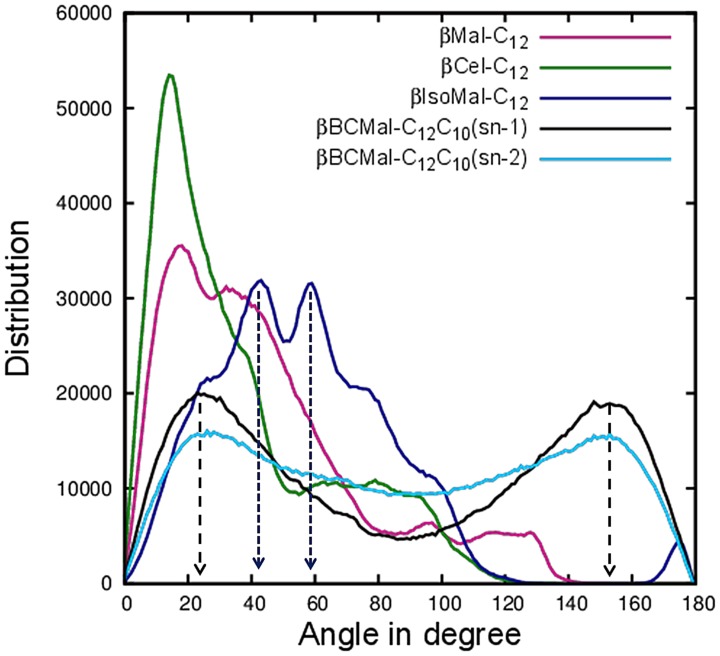
Distributions of alkyl chain tilt angle θ of various glycolipids for one layer. Shows the current results for 160*β*Mal-C_12_, black for *β*BCMal-C_12_C_10_ (*sn-1*), deep sky blue for *β*BCMal-C_12_C_10_(*sn-2*), green for *β*Cel-C_12_ and dark blue for *β*IsoMal-C_12_.

In general the non-reducing sugar (*ring2*) has slightly faster rotational diffusion than the reducing sugar (*ring1*) and the combined sugar headgroup (*ring12*). In particular for *β*IsoMal-C_12_, the non-reducing sugar's correlation time (τ*_ring2_*) on average is the smallest, which is consistent with the fact that this lipid has a smaller *d*-spacing and a larger *A* compared to the other glycolipid systems. Depending on the stereochemistry, the headgroup correlation time of the lipid headgroup differs only slightly such that, τ*_ring12_* of *β*Mal-C_12_ (with a α(1–4)-linkage) and *β*Cel-C_12_ (with an β(1–4)-linkage) is 78 and 80 ns respectively. The τ*_ring12_* values for *β*Mal-C_12_ and *β*IsoMal-C_12_ are also similar, despite the structural difference in the headgroup. The similarity in this dynamical behavior can be related to the distributions of hydrogen bonds in both *β*Mal-C_12_ and *β*IsoMal-C_12_. In both disaccharides, the major hydrogen bonding occurs at the hydroxymethyl group on the non-reducing sugar, such that the O6 acts as an acceptor while the HO6 acts as a donor. However, the next most dominant hydrogen bond site is O6' in *β*Mal-C_12_, but O1' in *β*IsoMal-C_12_ (see Figure 7: (a) and (d) from [22]). In *β*Mal-C_12_ the O6'-HO6' group (see Figure 1) acts as donor and acceptor, while in *β*IsoMal-C_12_ the O1' acts only as an acceptor. It was also found that the headgroup correlation time, τ*_ring12_*, for the branched glycolipid is shorter (hence faster rotational diffusion) compared to the monoalkylated one, even though both *β*Mal-C_12_ and *β*BCMal-C_12_C_10_ have the same sugar group. We note that the branched glycolipid exists in the L_α_ phase, where the constituent lipid chains tend to be more flexible, with many modes of motions, and the headgroup moiety is subjected to a greater motion due to the increased area per headgroup.

### Chain tilting

The chain tilting behavior was analysed by choosing a vector from the midpoints of C71–C72 and C81–C82 for the monoalkylated and the *sn-1* chains, while for the *sn-2* chain, it is defined from the midpoints of C83–C84 and C91–C92 (see Figure S1(b)). We plotted the distributions of these tilt angles using the first leaflet for the four systems (Figure 7). The maximum tilt values for *β*Cel-C_12_ and *β*Mal-C_12_ do not differ from the previous simulations [22], with values of 18° and 15° respectively (Table 1). Interestingly, for both the *β*IsoMal-C_12_ and *β*BCMal-C_12_C_10_, the maximum tilt values differ and the profiles display a more prominent bimodal distribution. In the case of isomaltoside, the two maxima are at 43° and 59° (Figure 7), which accounts for the significantly smaller *d*-spacing compared to the other two glycolipid systems considered here. A tilt angle θ of zero implies a perfect alignment of the chain vector with the layer normal, while a value of 180° implies the chain vector has “flipped” over. In the case of *β*IsoMal-C_12_, the two peak values represent the most probable chain vector orientations possibly arising from the α(1–6) glycosidic linkage connecting the two glucose units. In the previous simulation [22], these peaks were small, but are more populated in the current study; whilst this may arise simply from the additional sampling of this replicate, it may also be due to the greater freedom available to the system in using a fully anisotropic pressure scaling regime.

**Figure 8:**
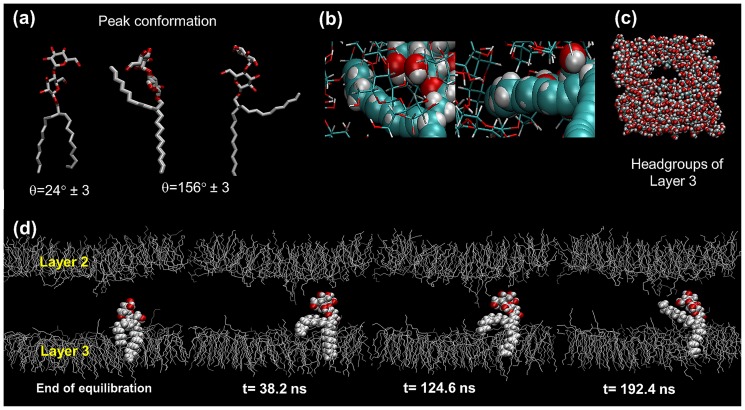
Some lipids' conformations and bilayer snapshots from the simulation. (a) Three possible lipid conformations with the chain tilt angle, θ of 24° and 156°. (b) The dispersion interaction observed from two viewing angles, between the C–H from the sugar face and those of the alkyl chain by a representative lipid (enlarged) from the third layer. (c) A characteristic example of the sugar headgroup region (t = 140 ns) from the third leaflet showing the hydrophobic cavity (top-view). (d) The time evolution of a typical side-view of the second and third leaflets. The lipid (drawn fully with VDW model) is seen to work itself into the hydrophilic region.

The branched chain glycoside is also bimodal in both the chain profiles. Firstly, the *sn-1* and *sn-2* chains have a non-zero population at the θ value of 90°, meaning some chains are parallel to the bilayer. In addition, there are double peaks symmetrical about the 90° angle for both the two branched chains, indicating there are two possible conformations equally populated at θ of 24° and 156°. To investigate this behavior and if it involves lipid flipping, we examined the glycolipid dynamics visually and found evidence of chain penetration into the hydrophilic headgroup region. As an example of this behavior, we present a movie of the last 160 ns production simulation, highlighting a lipid in the third leaflet (see Video S1 generated using VMD [51]). It is apparent over the course of the simulation that its chain works its way into the sugar headgroup environment. Based on the tilt angle distribution in Figure 7, we estimate for every leaflet there are about 10% of such lipids, which can be verified by examining the chain layer. We also present a movie focusing on the dynamics of the lipid headgroup region (Video S2); here we can see the emergence of the complementary hydrophobic cavity within the sugar region, which accommodates the lipid tail. From these dynamics visualization studies, we also discount the possibility of lipid flip. Nevertheless, by observing the movie and the way the chain vectors *sn-1* and *sn-2* are defined, these two peak conformations (θ of 24° and 156°) are possible as shown in Figure 8a, a snapshot captured from the movie. These results imply the chains can readily protrude into the hydrophilic region due to the formation of a “cavity” or “hole” in the headgroup region by sugar cooperative motion (Figure 8c), which is large enough to accommodate the alkyl chain. Figure 8d shows the time evolution of a selected lipid, whose alkyl chain made an attempt to associate itself with the headgroup region (represented by the wire and VDW model).

## Discussion

We report 160 ns simulations of four glycosides namely *β*Mal-C_12_, *β*Cel-C_12_, *β*IsoMal-C_12_ and *β*BCMal-C_12_C_10_ in anhydrous bilayer assemblies, to understand the relationship of structural and dynamical properties to the stereochemistry of these sugar based lipids. The stereochemistry of the sugar group is known to affect profoundly the assembly states and our work here has provided further evidence especially in the chain and headgroup orderings, structure and dynamics. For the three glycosides in the same L_C_ phase, the chains for *β*IsoMal-C_12_ are in a more disordered state compared to *β*Cel-C_12_ and *β*Mal-C_12_ (Figures 2 and 3). This is exemplified by the higher population of *gauche* chain dihedral conformations for *β*IsoMal-C_12_ compared to *β*Cel-C_12_ and *β*Mal-C_12_ (Figure 4). The lower ordering of the *β*IsoMal-C_12_ chains is most probably due to the increase in lateral area per lipid at the interface, which is related to the α(1–6) glycosidic linkage between two sugar moieties.

For the branched glycolipid, *β*BCMal-C_12_C_10_, which is in the L_α_ phase, a higher *gauche* population than for the monoalkylated lipid is observed although the systems are at the same temperature. Overall, this suggests the *gauche* population is influenced by chain branching and glycosidic linkage between two sugars, but less affected by the anomeric conformation or temperature. Consequently, the *gauche* populations affect some bilayer properties, including the bilayer *d*-spacing, as have been demonstrated experimentally from the study of anhydrous octyl *β*-maltoside (*β*Mal-C_8_) and decyl *β*-maltoside (*β*Mal-C_10_) bilayers using differential scanning calorimetry (DSC) and small angle x-ray scattering (SAXS) at ∼25°C in the L_C_ phase. In this study, the measured bilayer *d*-spacing was different before and after the glass transition *T_g_* [52]. The *gauche* population in the L_α_ phase is higher compared to the glass phase by 3% and 7% for *β*Mal-C_8_ and *β*Mal-C_10_ respectively [52]. On cooling from the L_α_ phase into the glassy phase, both the systems retained their liquid crystalline chain ordering, because a longer time was needed for the chains to regain the all-*trans* conformation [52]. In addition, long chain alkyl maltosides, such as tetradecyl *β-*maltoside (*β*Mal-C_14_) and hexadecyl *β-*maltoside (*β*Mal-C_16_) in the anhydrous state have been studied [18]; both systems have the same melting temperature of about 105°C, but phases below this temperature are complex and strongly driven by kinetics, causing them to be metastable. The lamellar structure in the anhydrous low temperature crystal has a *d*-spacing of about 37 Å but in the anhydrous high temperature crystal, the inter-lamellar distance is 43 Å. The difference of 6 Å (or 14%) could be attributed to the chain tilting together with the presence of an all-*trans* conformation in the anhydrous low temperature crystal (Figure S4). Meanwhile, the liquid crystalline state has a *d*-spacing about 40–41 Å (summary given in Table S2), where the value is closer to the *d*-spacing in the glass phase (Figure S4). But the ordered lamellar phase, which has the all-*trans* conformation for the chains, has about 7% less than the *d*-spacing (38 Å) compared to the L_α_ phase, which may be attributed to the tilted chain with respect to the bilayer normal. The presence of *gauche* conformations in the L_α_ phase reduces interdigitation, leading to a higher *d*-spacing relative to the other bilayer phases, except for the anhydrous high temperature crystal phase.

The sampling of chain conformations for the monoalkyl glycolipids in the lamellar crystalline phase, L_C_, is further reflected by analysis of rotational diffusion (Figure 5). These lipid chains may interact non-specifically [53], where the carbon atoms in the chains are able to rotate in a limited fashion within the lattice, leading to partial local rotations along the chain, as observed previously in the L_β_ or the gel phases for example [54]. Although *β*Mal-C_12_ and *β*BCMal-C_12_C_10_ have the same sugar headgroup, the carbons along the chain from both systems show different rotational behavior (Figure 5). This arises from the steric constraints of the two branching chains in *β*BCMal-C_12_C_10_; the resulting ‘chain-overcrowding’ in the hydrophobic region leads to the dominance of a repulsive van der Waals interaction. From our analysis of the autocorrelation function and visual inspection (Video S2), we note that the sugar headgroups also experience some partial “rotational motion”, although much slower than the chain carbon partial rotation. Before leaving the subject on dynamics, we comment on one of the *β*IsoMal-C_12_ stereoisomers, namely the gentibioside, whose non-reducing sugar is attached to the reducing sugar by a *β*(1∼6) linkage. The bilayer L_α_ phase of this gentibioside was studied using a ^2^H-NMR by Carrier *et al*. [55], who found its headgroup dynamics is relatively slower compared to other glycolipids studied at that time. Qualitatively this finding perhaps could lend support for the observed difference in the dynamics of the isomaltose headgroup as compared to the other sugar lipids.

For phospholipid systems, there are extensive studies of headgroup dynamics focused specifically on the hydrophilic moiety's rotational diffusion, albeit mainly in aqueous solution [50], [56], [57]. Many phospholipid headgroups act only as hydrogen bonding acceptors [58] but the sugar groups in glycolipids are both acceptors and donors of hydrogen bonding. This property of the headgroup grants glycolipids a strong coupling with its neighboring lipid headgroups in the self-assembly. Moreover, the covalent framework of the sugar, containing aligned C–H groups as well as polar groups, leads to its amphoteric nature [59]–[61]. This important property of sugars increases the intra-layer hydrogen-bond interaction, self-assembly stability and makes the phase boundary temperature independent [62]. So the headgroup motion is highly restricted by the extensive hydrogen bonds with the neighboring lipids. A comparison of the studies of phospholipid and glycolipid surfactants under thermotropic conditions, by Huang and Li [63] and Auvray *et al*. [19] respectively, further illustrates the distinctive behavior of sugar lipids. In these studies, phosphatidylcholine lipids were reported to undergo a gel to liquid-crystalline transition at about 70°C, while, at nearly the same temperature, glycolipids such as *β*Mal-C_12_ experienced a solid˜ solid phase transition, followed by a gel to liquid-crystalline phase transition at ∼103°C. Ericsson *et al*. [18] has suggested that the melting of alkyl-maltosides (from lamellar crystal to a liquid crystal phase) is primarily governed by the nature of the headgroup rather than by the alkyl chain length, primarily due to the extensive inter-molecular hydrogen bonding within the headgroup.

Conventionally, the association of sugar headgroups with lipophilic alkyl tails is considered to be highly unfavourable. However, the amphoteric nature of the sugar headgroups here has made this possible: the lipid chain can associate itself with the hydrophobic face of the saccharide unit [64] (see Figure 8b). There are many examples of saccharide binding to aromatic groups, e.g. with the Tyr, Phe and Trp amino acid residues of proteins [65], [66]. Our observation of chain-sugar association is not unreasonable, since other studies have appeared in recent years (e.g. [67]). The formation of hydrophobic cavities may provide the seeding condition for lipid flip-flop, a rare event, which may possibly be observed in a much longer simulation, even in an anhydrous bilayer system. A MD simulation which demonstrated lipid flip, performed by Gurtovenko and Vattulainen [68], suggested that the formation of a water hole in the hydrophobic region is an enabling condition for the flip motion. Our results point to the effects of chain crowding in forcing some lipids to protrude into the hydrophilic region. It is interesting to consider the possibility that, as these lipid tails vacate the hydrophobic cavity, the defects can permit seepage of water (if present) into these regions, potentially forming a pore for water. Therefore, our anhydrous branched chain glycolipid system, uncomplicated by the presence of solvent, may give insights into the formation of water defects in glycolipids [69].

## Conclusion

Glycolipids, although present in a smaller quantity than phospholipids, are ubiquitous in cell membranes and exhibit a diverse range of structures. This suggests they are important in cell functions, for example, in cell aggregation and dissociation. A glycolipid is classified as an amphitropic liquid crystal, able to self-assemble in a dry or solvated environment through separation of the extensively hydrogen bonded hydrophilic region from that of the repulsive hydrophobic alkyl chain. Systematic examination of the structure and dynamics as a function of sugar stereochemistry and hydrocarbon chain is necessary to understand the complex organization within this self-assembly system. Complementing our earlier structural studies of these systems, we report a dynamical analysis of the three monoalkylated glycosides with maltose, cellobiose and isomaltose sugar headgroups in the lamellar crystal (L_C_) phase and a branched chain maltoside in the lamellar (L_α_) phase. Our simulations suggest that chain packing is not influenced significantly by the anomeric configuration at the glycosidic linkage that connects two headgroups. Thus, the *β*(1–4) linked *β*Cel-C_12_ has a similar packing to the α(1–4) linked *β*Mal-C_12_. On the other hand, the RDF of the *α*(1–6) link of *β*IsoMal-C_12_, is in a marked contrast to these two, implying it has a more loosely packed structure. In the current study, where the lipids are at the room temperature, the chains do sample a small amount of *gauche* conformation. This suggests the L_C_ phase of these glycolipids is not entirely in a solid phase but possesses some chain disorder. We also observed the rotational dynamics of the headgroup and tail exhibit different behavior, where the headgroup rotates slower than the tail by a factor of at least two. Our simulations also suggest an unusual feature for the anhydrous bilayer system of branched chain glycolipids; in this system with its overcrowded chain region, the alkyl chains work themselves into the headgroup region and appear to associate with the more hydrophobic face of the sugar. Further work is necessary to explore the generality of these alkyl chain saccharide headgroup interactions for a range of glycolipid systems. Nevertheless, together with the emerging concepts of lateral segregation, lipid flip and domain formation, these findings provide new insights into our understanding of membrane stability and integrity.

## Supporting Information

Figure S1
**Definition of vector** 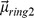 **from C1 to C4, vector** 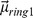 **C1' to C4' and** 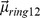 **from C1' to C4 for the sugar groups.** The chain vector 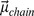 (for all monoalkylated lipids) and 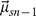 (for *sn*-1 chain) are defined from the mid points between C71–C72 and C81–C82. For *sn*-2 chain, the vector 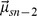 is defined from the mid points between C83–C84 and C91–C92.(TIF)

Figure S2 **Area per lipid of** ***β*** **Mal-C_12_,** ***β*** **Cel-C_12_,** ***β*** **IsoMal-C_12_, and** ***β*** **BCMal-C_12_C_10_.**
(TIF)

Figure S3 **Local density profile. Each plot comprises 40 ns blocks of averages.(a)** ***β*** **Mal-C_12_, (b)** ***β*** **Cel-C_12_, (c)** ***β*** **IsoMal-C_12_, (d)** ***β*** **BCMal-C_12_C_10_(** ***sn-1*** **) and (e)** ***β*** **BCMal-C_12_C_10_(** ***sn-2*** **).**
(TIF)

Figure S4 **Schematic representations of the phase structures characterized for tetradecyl** ***β-*** **maltoside (** ***β*** **C_14_G_2_).** (Redrawn from *Er*i*csson et al*.[18])
(TIF)

Figure S5 **Correlation functions for each C–H vector along lipid alkyl chains are shown.** The legend shows the carbon atoms following the numbering in Figure 1.
(TIF)

Figure S6
**Second rank reorientational autocorrelation functions** 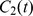 **for the sugars at the headgroup region for all the four glycosides, namely,** ***β*** **Mal-C_12_,** ***β*** **Cel-C_12_,** ***β*** **IsoMal-C_12_, and** ***β*** **BCMal-C_12_C_10_ for (a) non-reducing sugar (** ***ring1*** **), (b) reducing sugar (** ***ring2*** **) and (c) both the sugars together (** ***ring12*** **).**
(TIF)

Table S1 **Dihedral angles (in degrees) for glycosides at the glycosidic bond between the two sugar units.**
(DOC)

Table S2 **Phase transition temperatures for glycosides in an anhydrous condition from literatures.** The lamellar distance of bilayers, *d* (in Å).
(DOC)

Video S1 **A movie showing the last 160 ns production, focusing only on one lipid (VDW model) in the third leaflet which tries to burrow one of its chains into the hydrophilic region in the simulation box.** The head group region is represented by the wire frame model.
(AVI)

Video S2 **A movie of the top view of the lipid headgroup region for 160 ns.** For clarity the hydrophobic part has been removed to show the hydrophobic cavity within the headgroup.
(AVI)
