# Supplementary figures and images for: Conformational Dynamics of Dry Lamellar Crystals of Sugar Based Lipids: An Atomistic Simulation Study
Source: PLoS One. 2014 Jun 30;9(6):e101110. doi: 10.1371/journal.pone.0101110 (PMC4076255; doi:10.1371/journal.pone.0101110)

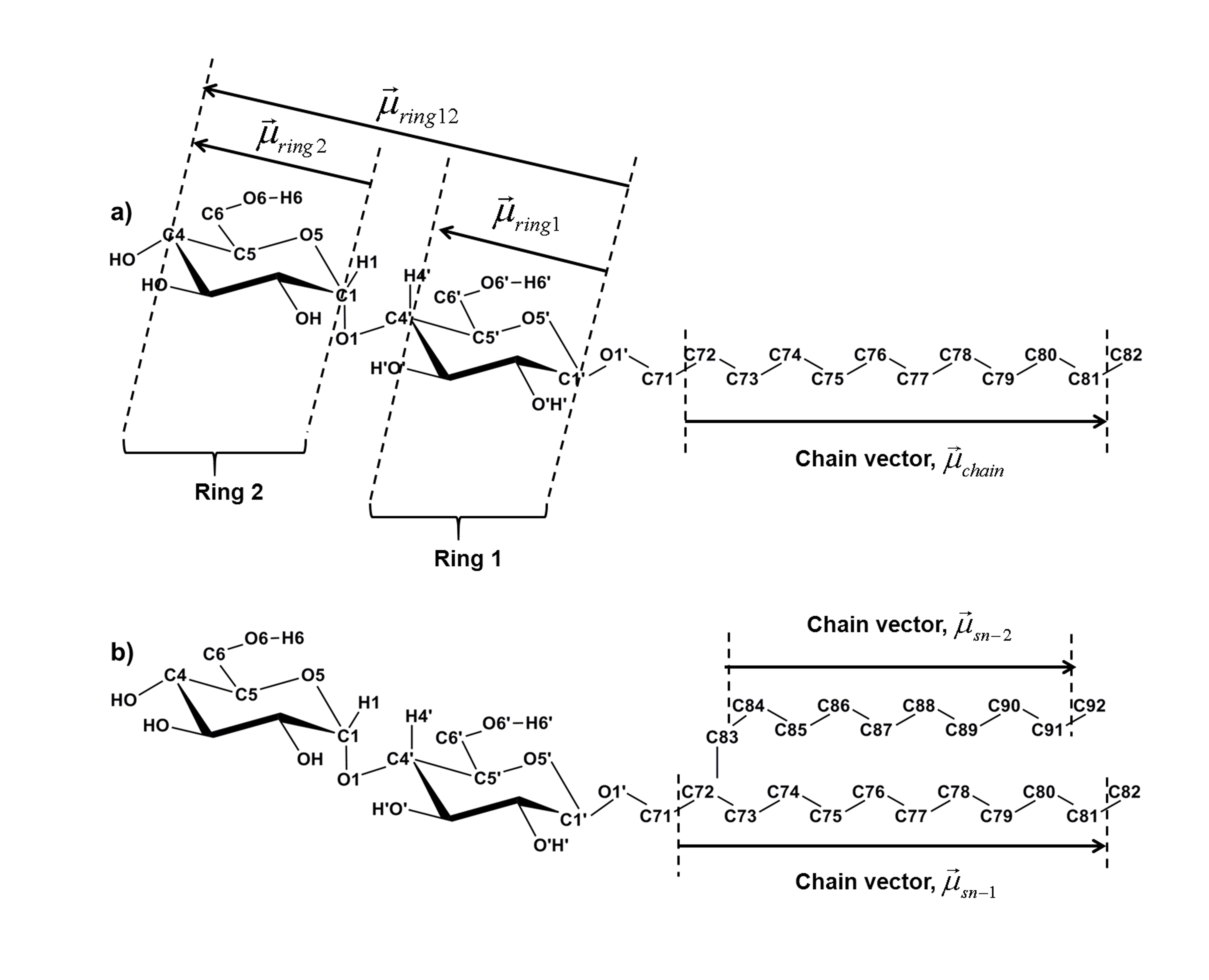

Supplement: Figure S1 — Definition of vector from C1 to C4, vector C1' to C4' and from C1' to C4 for the sugar groups. The chain vector (for all monoalkylated lipids) and (for sn-1 chain) are defined from the mid points between C71–C72 and C81–C82. For sn-2 chain, the vector is defined from the mid points between C83–C84 and C91–C92. (TIF) [file pone.0101110.s001.tif]

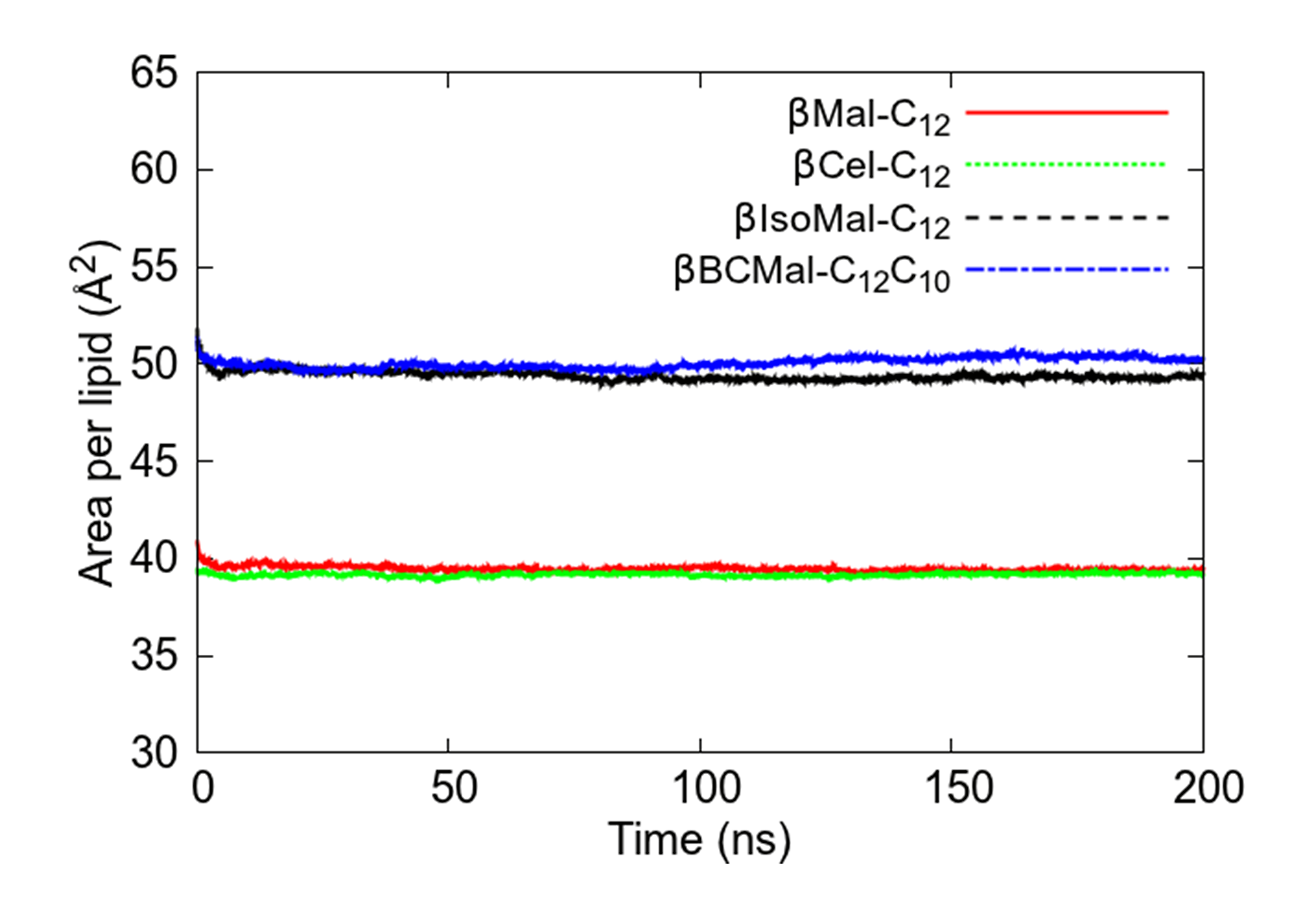

Supplement: Figure S2 — Area per lipid of β Mal-C12, β Cel-C12, β IsoMal-C12, and β BCMal-C12C10. (TIF) [file pone.0101110.s002.tif]

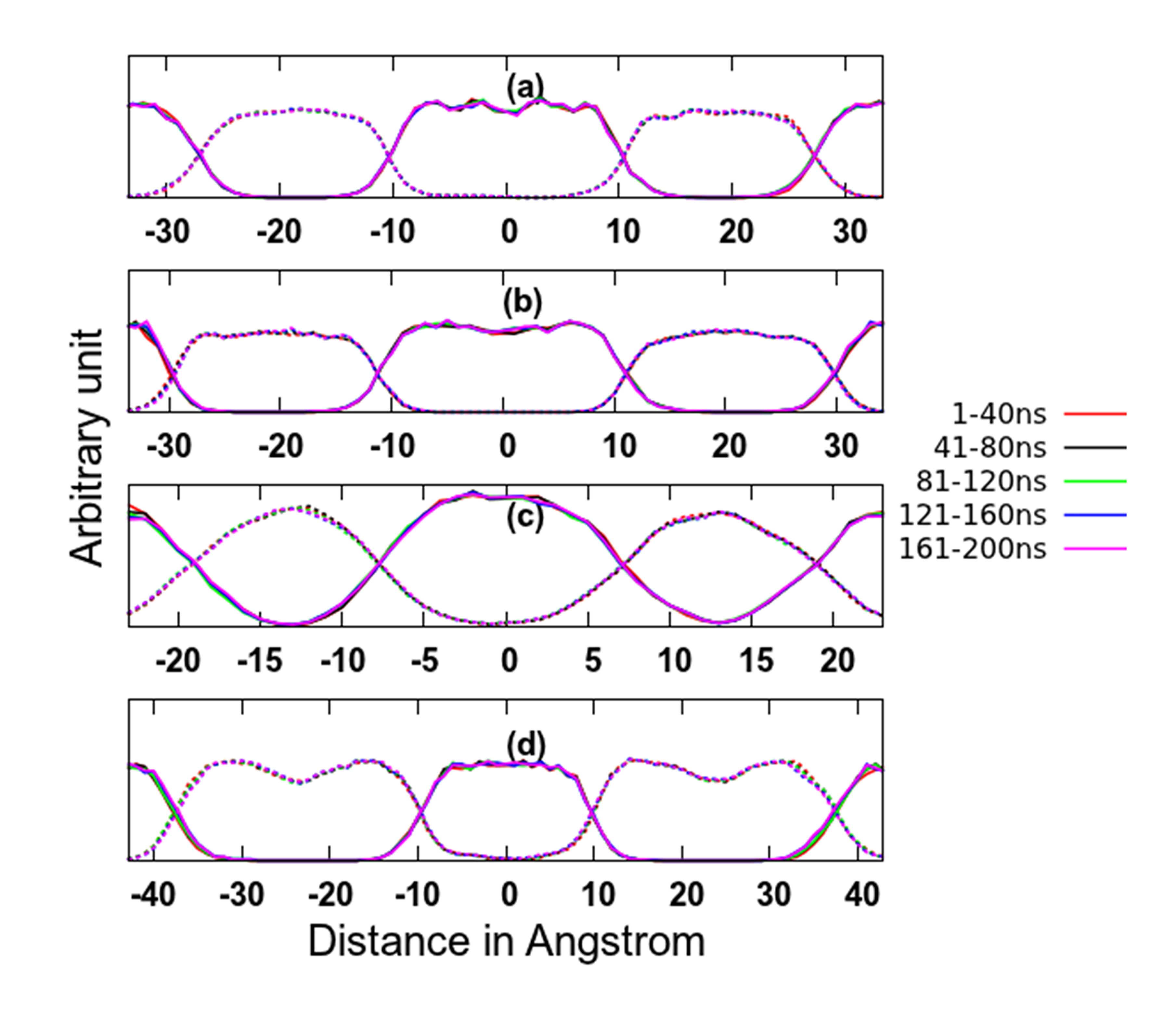

Supplement: Figure S3 — Local density profile. Each plot comprises 40 ns blocks of averages.(a) β Mal-C12, (b) β Cel-C12, (c) β IsoMal-C12, (d) β BCMal-C12C10( sn-1 ) and (e) β BCMal-C12C10( sn-2 ). (TIF) [file pone.0101110.s003.tif]

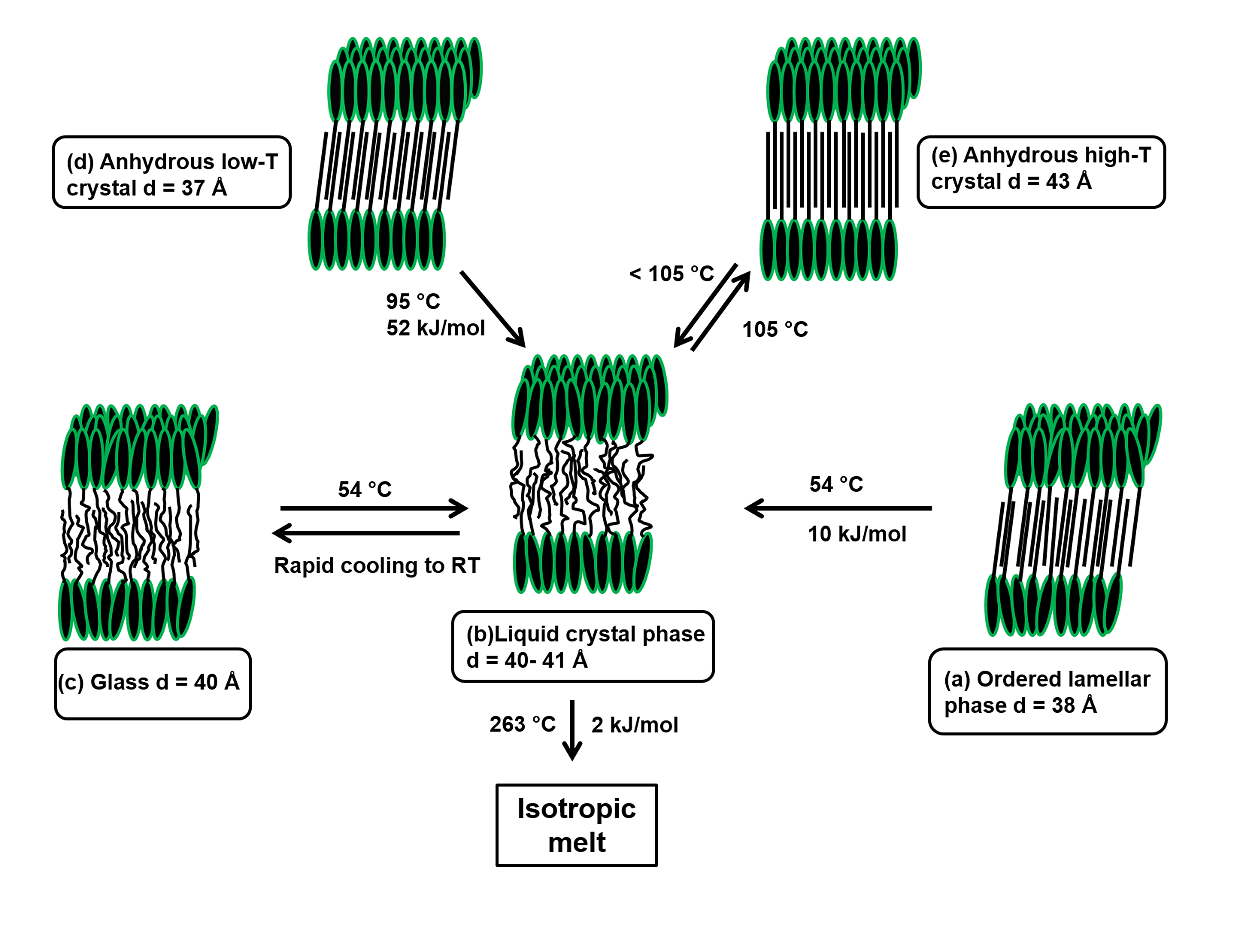

Supplement: Figure S4 — Schematic representations of the phase structures characterized for tetradecyl β- maltoside ( β C14G2). (Redrawn from Ericsson et al.[18]) (TIF) [file pone.0101110.s004.tif]

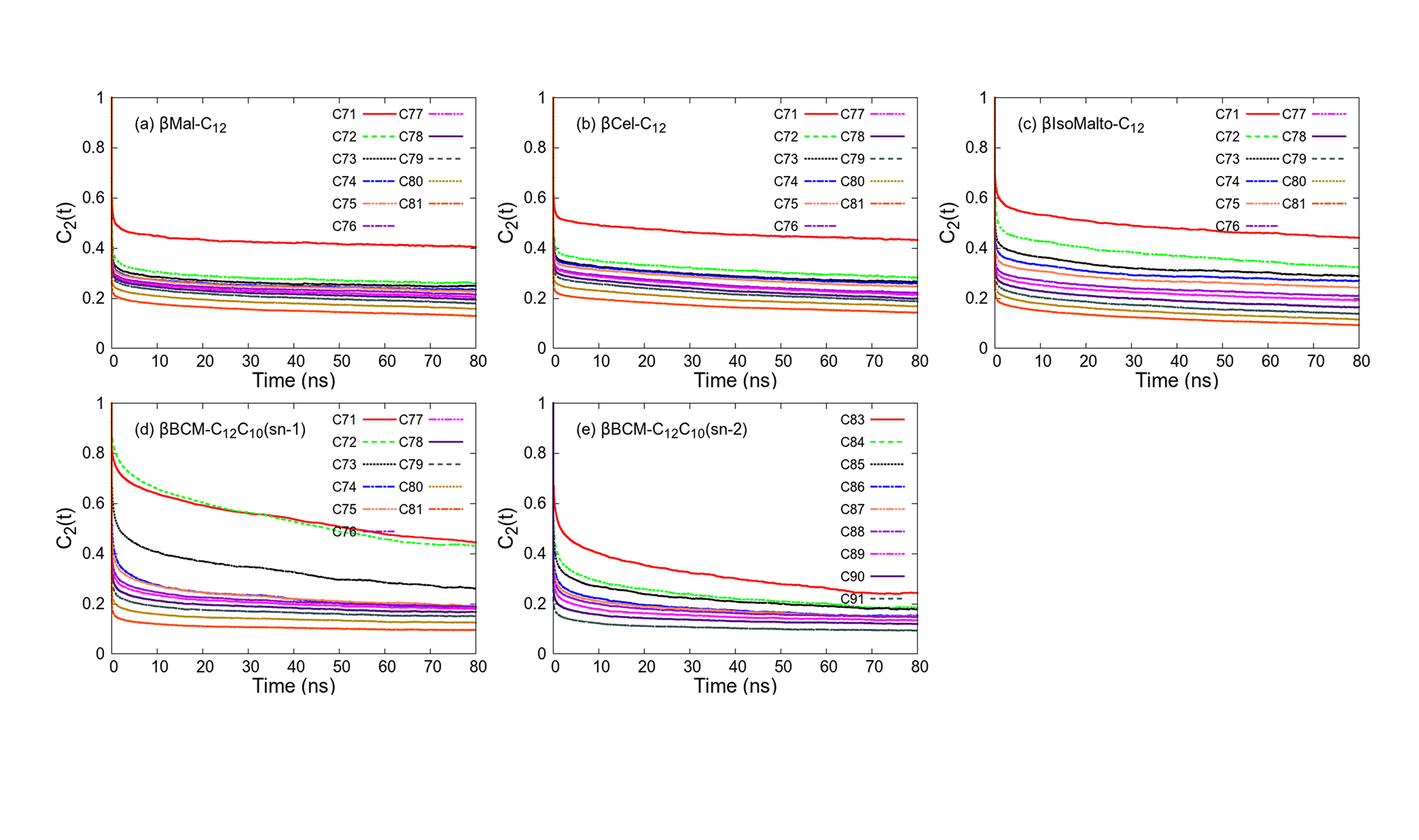

Supplement: Figure S5 — Correlation functions for each C–H vector along lipid alkyl chains are shown. The legend shows the carbon atoms following the numbering in Figure 1. (TIF) [file pone.0101110.s005.tif]

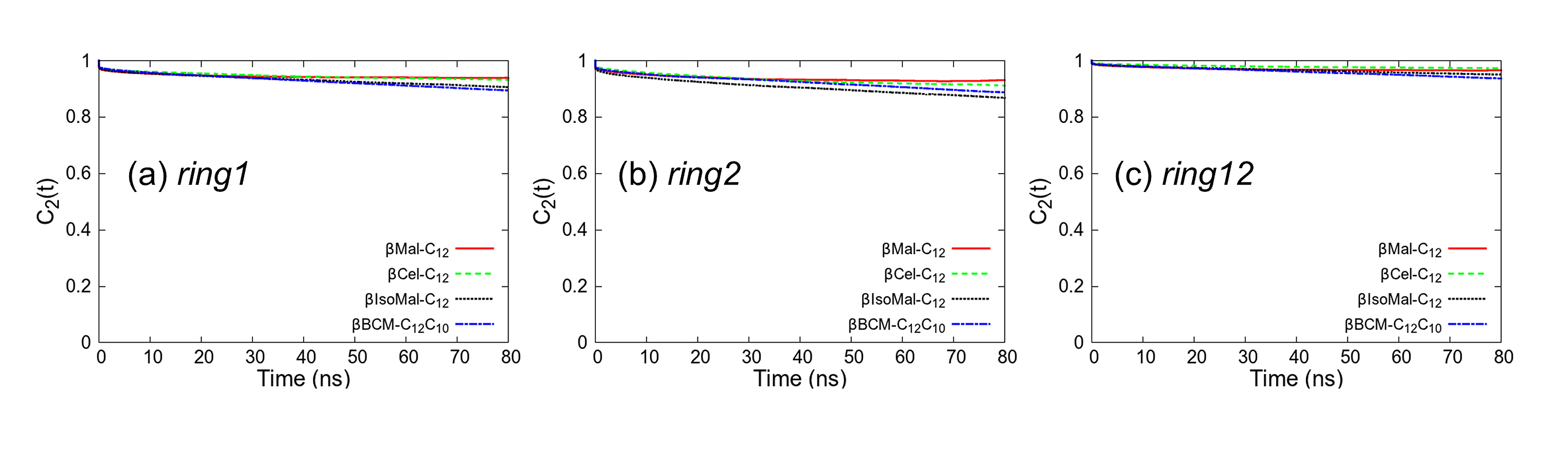

Supplement: Figure S6 — Second rank reorientational autocorrelation functions for the sugars at the headgroup region for all the four glycosides, namely, β Mal-C12, β Cel-C12, β IsoMal-C12, and β BCMal-C12C10 for (a) non-reducing sugar ( ring1 ), (b) reducing sugar ( ring2 ) and (c) both the sugars together ( ring12 ). (TIF) [file pone.0101110.s006.tif]
