# Supplementary material for: Conformational Dynamics of Dry Lamellar Crystals of Sugar Based Lipids: An Atomistic Simulation Study
Source: PLoS One. 2014 Jun 30;9(6):e101110. doi: 10.1371/journal.pone.0101110 (PMC4076255; doi:10.1371/journal.pone.0101110)
Supplement: Table S1 — Dihedral angles (in degrees) for glycosides at the glycosidic bond between the two sugar units. (DOC) [file pone.0101110.s007.doc]

| Table S1. Dihedral angles (in degrees) for glycosides at the glycosidic bond between the two sugar units. | | | | |
| --- | --- | --- | --- | --- |
|  | Φ (H1-C1-O1-C4') | ψ(C1-O1-C4'-H4') |  | Ref |
| *β*Mal-C_12_ | -36.0 | -49.0 | - | [25] |
| *β*Cel-C_12_ | 48.0 | -25.0 | - | [23] |
|  | Φ(O5-C1-O1-C6') | ψ(C1-O1-C6'-C5') | ω(O1-C6’-C5’-C4’) |  |
| *β*IsoMal-C_12_. | -49.2 | 167.3 | -45.3 | [26] |
