# Supplementary material for: Conformational Dynamics of Dry Lamellar Crystals of Sugar Based Lipids: An Atomistic Simulation Study
Source: PLoS One. 2014 Jun 30;9(6):e101110. doi: 10.1371/journal.pone.0101110 (PMC4076255; doi:10.1371/journal.pone.0101110)
Supplement: Table S2 — Phase transition temperatures for glycosides in an anhydrous condition from literatures. The lamellar distance of bilayers, d (in Å). (DOC) [file pone.0101110.s008.doc]

| **Table S1.** Phase transition temperatures for glycosides in an anhydrous condition from literatures. The lamellar distance of bilayers, d (in Å). | | | | | | | | | |
| --- | --- | --- | --- | --- | --- | --- | --- | --- | --- |
| Lipid | Symbol | *d-*spacing/ Å | Pre-transition temp | | | | Solid–Lα | Lα–iso. | Ref |
|  |  | ( at 20°C) | (°C) | | | | transition temp | transition temp. |  |
|  |  |  |  | | | | (°C) | (°C) |  |
|  |  |  |  |  |  |  | (*d-*spacing) |  |  |
| Octyl *β*-glucoside | *β*C8­Glu |  |  |  |  |  | 69.0 | 107.0 | [10] |
| Octyl *β*-maltoside | *β*C8Mal |  |  |  |  |  |  | 122.7 | [52] |
| Decyl *β*-maltoside | *β*C10Mal |  |  |  | 78.6(c) |  | 96.5 | 203.0 | [52] |
| Decyl *β*-maltoside | *β*C10Mal |  |  |  |  |  | 38.6 | 102.2 | [52] |
| Decyl *β*-maltoside | *β*C10Mal |  | 25(a) | 25(b) |  |  |  | 208.0 | [52] |
| Dodecyl *β*-maltoside | *β*C12Mal | 33.5 |  |  | 70(c) |  | 103.0 (41.5 Å) |  | [19] |
| Dodecyl *α*-maltoside | *α*C12Mal | 33.5 |  |  | 50(c) |  | 128.0 ( 32.4 Å) |  | [19] |
| Tetradecyl *β-*maltoside | *β*C14Mal |  |  |  |  | 95(d) | 105.0 (40-41 Å) | 263.0 | [18] |
| Hexadecyl *β-*maltoside | *β*C16Mal |  |  |  |  |  | 105.0 |  | [18] |
| Note: (a) glassy state before heating (b) glassy state after heating (c) solid/solid transition (d) not solid/solid transition. | | | | | | | | | |
